# Neuroprotective and anti-inflammatory activity of *Wyethia* species: therapeutic potential for neurodegenerative diseases

**DOI:** 10.3389/fphar.2025.1740121

**Published:** 2026-01-15

**Authors:** Idowu Jonas Sagbo, David Soriano-Castell, Jon Rebman, Pamela Maher

**Affiliations:** 1 Cellular Neurobiology Laboratory, Salk Institute for Biological Studies, La Jolla, CA, United States; 2 Department of Botany, San Diego Natural History Museum, San Diego, CA, United States

**Keywords:** Alzheimer’s disease, neuroprotection, oxytosis/ferroptosis, polyphenols, *Wyethia*

## Abstract

Neurodegenerative diseases such as Alzheimer’s disease involve regulated forms of cell death, including oxytosis/ferroptosis, which are driven by oxidative stress and neuroinflammation. Targeting these regulated cell death pathways offers novel therapeutic opportunities. *Wyethia* is a small genus of flowering plants native to North America, traditionally used by Indigenous populations for medicinal purposes. Its ethnobotanical relevance and phytochemical diversity prompted investigation into its neuroprotective potential. This study examined eight *Wyethia* species for anti-inflammatory and neuroprotective activities. We evaluated protection against oxytosis/ferroptosis (induced by glutamate, erastin, and RSL3) in HT22 neuronal cells, energy loss using an ischemia assay in HT22 cells, and LPS-induced inflammation in BV2 microglial cells. *W. ovata*, *W. helenioides*, *W. amplexicaulis* and *W. glabra* exhibited strong neuroprotection (EC_50_ < 25 μg/mL), with *W. ovata* also demonstrating potent anti-inflammatory activity (EC_50_ = 15.2 μg/mL). Antioxidant assays (DPPH, lipid peroxidation) and measurement of total phenolic content (TPC) analysis revealed a correlation between high phenolic content and antioxidant activity, with *W. ovata* and *W. amplexicaulis* showing the highest TPC values (120–140 mg GAE/g). However, *W. invenusta* displayed strong neuroprotective effects despite low TPC, suggesting that other bioactive compounds such as terpenoids, or alkaloids may contribute to its activity. This highlights the complexity of phytochemical profiles and the potential presence of potent constituents beyond phenolics. These findings position *Wyethia* as a promising source of dual-action neuroprotective agents that target both oxytosis/ferroptosis and inflammation, warranting further phytochemical investigation.

## 1 Introduction

Neurodegenerative diseases such as Alzheimer’s disease (AD) are characterized by progressive neuronal loss, leading to cognitive and motor impairments (Moya-Alvarado et al., 2016). An increasing number of studies indicate that iron-dependent oxidative cell death pathways, particularly oxytosis/ferroptosis, play a significant role in driving neurodegeneration in AD (Zhao et al., 2023; Zeng and Jin, 2025; Currais et al., 2025). These pathways are distinguished by mitochondrial dysfunction, lipid peroxidation (LPO) and glutathione (GSH) depletion, which together amplify neuroinflammation and synaptic toxicity (Tönnies and Trushina, 2017; Misrani et al., 2021).

In AD, dysregulated iron metabolism and impaired cystine/glutamate antiporter (xc^−^) activity exacerbate GSH depletion, rendering neurons vulnerable to oxytotic/ferroptotic death through uncontrolled lipid peroxidation (Soriano-Castell et al., 2021a; Sutanto et al., 2025). This vulnerability is compounded by overproduction of mitochondrial reactive oxygen species (ROS), which not only damages cellular components but also promotes amyloid-β (Aβ) aggregation and tau hyperphosphorylation, thereby perpetuating a vicious cycle of oxidative damage and neuroinflammation (Wang et al., 2023; Alkhalifa et al., 2025). Oxytosis/ferroptosis represents a form of regulated cell death that includes the iron-dependent accumulation of lipid hydroperoxides and the failure of antioxidant defenses (Chen et al., 2021). Glutamate and erastin induce this cell death pathway by inhibiting cystine uptake via the cystine/glutamate antiporter, leading to glutathione depletion (Soriano-Castell et al., 2021a). RSL3 triggers oxytosis/ferroptosis by directly inhibiting glutathione peroxidase 4 (GPX4), resulting in lethal lipid peroxidation. Given the mechanistic specificity of oxytosis/ferroptosis, therapeutic strategies that restore GSH levels, boost antioxidant defenses, modulate lipid peroxidation, or chelate iron are gaining traction for their potential to provide effective neuroprotection against the multifactorial pathologies of AD (Maher et al., 2020a; Sun et al., 2025).

Medicinal plants with the ability to regulate oxidative cell death pathways such as oxytosis/ferroptosis are gaining attention as promising neuroprotective agents (Maher et al., 2020a). Among these, species rich in polyphenols and flavonoids such as *Centella asiatica*, and *Curcuma longa* (turmeric) have been reported to inhibit microglial activation, scavenge ROS, and suppress LPO (Lee et al., 2017; Maher et al., 2020b; Hambali et al., 2024). For example, *Curcuma longa* extracts (containing curcuminoids and synergistic compounds) inhibit Aβ aggregation and tau phosphorylation in preclinical models (da Costa et al., 2018; Oliveira and Pieniz, 2024). However, many plant species with therapeutic potential remain underexplored, highlighting the need to investigate novel botanical sources of bioactive compounds.

The genus *Wyethia* (Asteraceae family), native to western North America, encompasses species traditionally used by Indigenous peoples for medicinal purposes. These include treatments for respiratory, gastrointestinal and dermatological ailments (Moore and Bohs, 2003). These plants, colloquially termed mule’s ears, contain phenolic acids, flavonoids, and sesquiterpene lactones, phytochemicals linked to anti-inflammatory and antioxidant effects in related Asteraceae species (Borgo et al., 2024). This ethnobotanical knowledge aligns with modern findings linking these phytochemicals to antioxidant and neuroprotective activities, thereby bridging traditional practice and laboratory investigation. *Wyethia angustifolia* and *W. helenioides* produce flavonoids, including quercetin derivatives, which are linked to ROS scavenging and NF-κB inhibition (McCormick et al., 1986). These phytochemicals may function through distinct mechanisms to disrupt oxytosis/ferroptosis at multiple points. These include iron chelation to inhibit LOX-driven lipid peroxidation, preservation of glutathione (GSH) via stabilization of the cystine/glutamate antiporter (xc^−^), and direct scavenging of reactive oxygen species (ROS) to prevent mitochondrial and membrane damage. Despite the rich ethnobotanical history of *Wyethia*, the potential of its species to modulate oxytosis/ferroptosis, and neuroinflammation in AD-relevant cell types remains largely unexplored. Traditional preparations of these plants often utilize leaves or roots, although detailed documentation of extraction methods is limited. In this study, dichloromethane extracts were used to target bioactive compounds with similar polarity.

In this context, we evaluated select *Wyethia* extracts for their ability to mitigate oxytosis/ferroptosis in HT22 hippocampal neuronal cells and for their anti-inflammatory effects in BV2 microglial cells. We also assessed their antioxidant capacity along with effects on GSH levels and lipid peroxidation to elucidate the mechanisms underlying their neuroprotective potential. Notably, several *Wyethia* extracts significantly protected HT22 cells against ferroptotic cell death, reduced oxidative stress by preserving GSH and lowering lipid peroxidation, and suppressed neuroinflammatory responses in BV2 cells. By addressing multiple age-associated toxicities relevant to AD, these findings highlight *Wyethia* species as promising botanical candidates for therapeutic intervention in neurodegenerative diseases. Furthermore, this study reaffirms the critical importance of herbarium collections as an excellent source of material for ethnopharmacological research. Herbarium specimens offer significant technical advantages over traditional field collection, including comprehensive historical documentation and accessibility, which were previously demonstrated in our work with the San Diego Natural History Museum. These advantages enable more robust and reproducible investigations, particularly when exploring traditional knowledge in botanical drug discovery.

## 2 Materials and methods

### 2.1 Plant collection and extraction

Eight species of *Wyethia* were obtained from authenticated specimens stored in the SD Herbarium at the San Diego Natural History Museum (SDNHM). These species were selected for study due to their ecological significance, the limited prior research on their neuroprotective activity and phytochemical properties, and their traditional use by indigenous peoples for treating various ailments, highlighting their potential relevance to understanding plant adaptation and medicinal applications. The leaves of these plants were utilized for the study. The identification and verification of the specimens were primarily conducted by Jon Rebman, SDNHM Curator of Botany. Descriptive information for the eight *Wyethia* taxa, including the accession number, species name, common name, and collector ID is described in Table 1. Permission for sampling these herbarium specimens for research purposes was granted by Rebman, the curator in charge of this collection.

**TABLE 1:** Descriptive information for *Wyethia* taxa studied.

SD accession number	Species	Common name	Collector ID	Date of collection
202836	*Wyethia scabra* Hook.	Mule’s Ears	Hodgson 9947	13 June 1996
171034	*Wyethia ovata* Torr. & A.Gray	Southern Mule’s Ears	Spears 237	1 June 2005
249157	*Wyethia helenioides,* Nutt.	Gray Mule’s Ears	Keil 20558-1	3 May 1988
69964	*Wyethia glabra* A.Gray	Coast Range Mule’s Ears	Howe 4522	23 May 1968
154101	*Wyethia elata* H.M.Hall	Hall’s Mule’s Ears	Adkins s.n.	20 May 2003
144483	*Wyethia invenusta* (Greene) W.A.Weber	Coville’s Mule’s Ears	Charlton 3716	21 July 1989
213682	*Wyethia amplexicaulis* (Nutt.) Nutt.	Northern Mule Ears	Lukas 11170	24 August 2009
131133	*Wyethia mollis* A.Gray	Woolly Mule’s Ears	Breedlove 62658	5 June 1986

Mature leaves from dried plant specimens were carefully collected from herbarium samples with sufficient material. The extraction process followed the same methods previously described (Maher et al., 2020b). Briefly, the dried leaves were crushed, and dichloromethane (DCM) extracts were prepared by weighing 100 mg of plant material and dissolving it in 1 mL of DCM. DCM has historically been used in our laboratory for the initial broad-scale screening of herbarium specimens due to its effectiveness in extracting lipophilic compounds such as flavonoids, sesquiterpene lactones and other non-polar constituents, which we have found are strongly associated with neuroprotective and anti-inflammatory activities (Borgo et al., 2024). Preliminary extractions were also conducted using aqueous solvent to mimic traditional preparations, but these extracts showed minimal neuroprotective activity in our assays. Based on these findings, and to focus on bioactive lipophilic compounds, DCM was selected as the solvent for the detailed analysis. The mixture was shaken overnight using a mechanical shaker and then left to stand at room temperature. The next day, the solution was filtered through Whatman No. 1 filter paper, and the resulting filtrate was concentrated to dryness using a rotary evaporator. Any residual DCM was completely evaporated in a fume hood overnight. Once fully dried, the extract was weighed to determine the yield (Table 2). The dried extract was then dissolved in dimethyl sulfoxide (DMSO) at a concentration of 50 mg/mL. Insoluble materials were removed by high-speed centrifugation and discarded. The final samples were stored at −80 °C for future use.

**TABLE 2:** EC_50_ values of *Wyethia* species extracts for neuroprotective and anti-inflammatory activities in HT22 and BV2 cells.

Plant name	SD accession	Percentage yield (%)	Toxicity (EC_50_, µg/mL)	Glutamate (EC_50_, µg/mL)	Erastin (EC_50_, µg/mL)	RSL3 (EC_50_, µg/mL)	IAA (EC_50_, µg/mL)	Anti-inflam. (EC_50_, µg/mL)
*W. scabra*	202836	3.2	>50	44.5	>50	>50	Not tested	Not tested
*W. ovata*	171034	5.5	6.2	2.2	3.3	2.5	3.4	13.5
*W. helenioides*	249157	10.7	>50	5.7	9.6	9.7	11.1	30.3
*W. glabra*	69964	14.9	>50	10.3	13.7	10.2	10.1	41.2
*W. elata*	154101	2.7	>50	No protection	>50	>50	Not tested	Not tested
*W. invenusta*	144483	1.7	>50	17.6	22.9	23.3	15.6	40.0
*W. amplexicaulis*	213682	6.1	>50	10.9	10.0	22.1	11.8	>50
*W. mollis*	62658	3.3	>50	>50	>50	23	33.3	35.7

EC_50_ values for protection against each insult are presented for all *Wyethia* extracts tested. Glutamate (5 mM), erastin (500 nM), and RSL3 (250 nM) were used to induce oxytosis/ferroptosis in HT22 cells, while IAA (15 µM) was used to induce metabolic energy loss. “No protection” indicates that no measurable protective effect was detected within the tested concentration range, and “>50 μg/mL” denotes that the EC_50_ exceeded the highest concentration tested.

All EC_50_ values were calculated from full dose–response curves using nonlinear regression (four-parameter logistic, variable-slope) modelling in GraphPad Prism, based on at least three independent biological experiments. Full dose–response datasets are available from the authors upon request.

### 2.2 Cell culture condition

HT22 mouse hippocampal neuronal cells were cultured in high-glucose Dulbecco’s modified Eagle’s medium (DMEM) (Invitrogen, Carlsbad, CA, United States) with 10% fetal calf serum (FCS) (Gibco, United States) and incubated at 37 °C in a 10% CO_2_ atmosphere. Mouse BV2 microglial cells were cultured in low-glucose DMEM with 10% FCS and incubated under the same conditions.

### 2.3 Neuroprotection assays

#### 2.3.1 Oxytosis/ferroptosis assay

For the oxytosis/ferroptosis assay, HT22 cells (5 × 10^3^) were seeded into 96-well plates and cultured for 24 h. The medium was then replaced with fresh medium, and cells were treated with 5 mM glutamate, 500 nM erastin, or 250 nM RSL3, along with extracts at concentrations ranging from 0.5–50 μg/mL. After 24 h of treatment, cell viability was assessed using the MTT (3-(4, 5-dimethylthiazolyl-2)-2,5-diphenyltetrazolium bromide) assay (Liang et al., 2022), which measures mitochondrial metabolic activity as an indicator of cell viability. In the absence of neuroprotective compounds, more than 90% of the cells die under these conditions. Prior to adding the MTT reagent, the plates were examined microscopically to confirm that any observed effects were not due to interactions between the extracts and the assay chemistry.

#### 2.3.2 Protection against energy loss (*in vitro* ischemia assay)

HT22 cells were seeded into 96-well plates as described in the oxytosis/ferroptosis assay. After 24 h, the medium was replaced with fresh medium, and the cells were treated with 15 µM Iodoacetic acid (IAA), which induces 90%–95% cell death. After 2 h, the medium was aspirated and replaced with fresh medium containing the plant extracts at concentrations ranging from 0.5–50 μg/mL. After 24 h of treatment, cell viability was assessed using the MTT assay. The results were further confirmed by visual inspection of the wells.

#### 2.3.3 Anti-inflammatory assay

The anti-inflammatory assay was performed as described (Fischer et al., 2019). Briefly, mouse BV2 microglial cells were plated at a density of 5 × 10^5^ in 35 mm tissue culture dishes and incubated overnight. The following day, cells were treated with 50 ng/mL bacterial lipopolysaccharide (LPS) alone or in combination with plant extracts at concentrations ranging from 5 to 50 μg/mL. After 24 h of incubation, the culture medium was collected and centrifuged at 5,000 rpm for 1 min at room temperature to remove floating cells. A 100 µL aliquot of the supernatant was then mixed with 100 µL of Griess reagent in a 96-well plate. Following a 10-min incubation at room temperature, absorbance was measured at 550 nm using a microplate reader.

#### 2.3.4 Total glutathione (GSH) measurement

The total GSH assay was conducted according to the method previously outlined (Liang et al., 2022). Briefly, HT22 cells were plated at a density of 3 × 10^5^ in 35 mm tissue culture dishes and incubated overnight. After 24 h, the medium was replaced with fresh medium and the indicated concentrations of glutamate (5 mM) and plant extracts were added. Following the 24 h treatments, cells were scraped into ice-cold PBS and mixed with 10% sulfosalicylic acid. The samples were kept on ice for 10 min and then centrifuged at 14,000 rpm for 10 min at 4 °C. The supernatant was transferred to a new tube containing 24 µL of triethanolamine: PBS (1:1). Total GSH levels were determined using a recycling assay based on the reduction of 5,5′-dithiobis (2-nitrobenzoic acid) with glutathione reductase and NADPH. GSH levels were normalized to protein content, which was recovered from the acid-precipitated pellet by treatment with 0.2 N NaOH at 37 °C overnight and quantified using the BCA protein assay (Pierce, Rockford, IL, United States).

#### 2.3.5 DPPH free radical scavenging assay

The DPPH free radical scavenging assay was conducted to evaluate the antioxidant activity of selected *Wyethia* species using the method described (Pringle et al., 2021). Briefly, 5 μL of the sample was mixed with 120 μL of Tris-HCl buffer (50 mM, pH 7.4) and 120 μL of DPPH solution (0.1 mM in absolute ethanol) in a 96-well microtiter plate. The mixture was incubated for 20 min at room temperature in the dark to prevent light-induced degradation. After incubation, the absorbance was measured at 513 nm.

#### 2.3.6 Lipid peroxidation assay

A cell-free assay to measure inhibition of lipid peroxidation was performed using the method described previously (Soriano-Castell et al., 2021a). A mixture of STY-BODIPY (1.5 μM) and egg-PC liposomes (1 mM) was prepared in TBS (pH 7.4) and dispensed into an opaque 96-well plate. The plant extracts were then added to the well at a final concentration of 25 μg/mL. The plate was incubated for 30 min and subsequently mixed vigorously for 5 min. Autoxidation was initiated by adding V-70 (750 μM), followed by another 5 min of mixing. Fluorescence measurements were taken every 15 min at 37 °C using a SpectraMax M5 microplate reader with excitation/emission wavelengths set at 488/518 nm. The raw fluorescence data were converted to oxidized STY-BODIPY concentration ([ox-STY-BODIPY]) by normalizing the saturated fluorescence values of the control to the initial concentration of reduced STY-BODIPY (1.5 μM).

#### 2.3.7 Total phenolic content

The total phenolic content was quantified using a modified version of the Folin–Ciocalteu (F-C) colorimetric assay as outlined (Kovačević et al., 2023). Samples and standards were pipetted into a 96-well plate, followed by the addition of ultrapure water (milliQ® water, Merck, NJ, United States) and the F-C reagent, which was diluted 1:1 with water. After a 5-min incubation period, a saturated sodium carbonate (Na_2_CO_3_) solution (7.5% w/v) was added. The plate was then incubated for 30 min, and absorbance was measured at 765 nm using a SpectraMax M5 microplate reader. The results were calculated by interpolating the data against a gallic acid standard curve (0–100 μg/mL). The total phenolic content (TPC) was expressed as milligrams of gallic acid equivalents per gram of extract (mg GAE/g extract) and reported as mean ± standard error of the mean (SEM).

### 2.4 Statistical analysis

Data from at least three independent experiments were normalized, pooled, and analyzed using GraphPad Prism software (version 10). Statistical tests applied are indicated in the figure legends, and a significance threshold of *P* < 0.05 was used. The half-maximal effective concentrations (EC_50_ values) were determined from sigmoidal dose-response curves generated using GraphPad Prism 10.

## 3 Results

### 3.1 Overview of the screening process and initial species evaluation

Eight *Wyethia* species were initially screened for their protective effects against oxytosis/ferroptosis using HT22 cells. Those extracts exhibiting significant protection (EC_50_ < 50 μg/mL) were further evaluated in the *in vitro* ischemia assay, also in HT22 cells. All *Wyethia* species that demonstrated significant protection in the oxytosis/ferroptosis assay were tested for anti-inflammatory activity in BV2 microglial cells, a widely used model for studying neuroinflammation. These cells retain essential microglial functions such as phagocytosis and the ability to respond to inflammatory stimuli by secreting nitric oxide (NO) and pro-inflammatory cytokines (Tawbeh et al., 2023).

To achieve a more comprehensive characterization, all eight *Wyethia* species were also subjected to a series of biochemical assays. The DPPH free radical scavenging assay was used to assess antioxidant activity, the cell-free lipid peroxidation assay evaluated lipid oxidation resistance, and total phenolic content was measured to estimate polyphenolic composition. This integrative approach enabled a thorough exploration of potential correlations between protective effects and biochemical properties across all species, regardless of their performance in the initial oxytosis/ferroptosis screening.

### 3.2 Cytotoxicity and neuroprotection in oxytosis/ferroptosis models

To ensure the safety and effective dosing of *Wyethia* extracts, cytotoxicity assays were performed on HT22 hippocampal neuronal cells. This testing identifies concentrations that do not harm cell viability, providing a baseline for subsequent neuroprotection experiments. Most *Wyethia* species exhibited no significant cytotoxicity at the tested concentrations of 5, 12.5, 25 and 50 μg/mL (LD_50_ > 50 μg/mL), except for *W. ovata*, which showed a toxicity EC_50_ of 6.2 μg/mL, indicating high cytotoxicity (Table 2). These findings highlight substantial variability in cytotoxic profiles among *Wyethia* species, which is critical for optimizing therapeutic dosing in models of oxytosis/ferroptosis.

### 3.3 Neuroprotective effects of *Wyethia* extracts in oxidative stress models

To evaluate the neuroprotective potential of *Wyethia* species against oxytosis/ferroptosis, we screened the non-toxic extracts against glutamate (5 mM), erastin (500 nM) and RSL3 (250 nM) at concentrations of 5, 12.5, 25, and 50 μg/mL. These compounds mimic oxidative stress-related neuronal death relevant to neurodegenerative diseases (Fei and Ding, 2024). Due to its cytotoxicity at concentrations above 5 μg/mL, *W. ovata* was additionally tested at lower concentrations (0.5, 1.25, 2.5, and 5 μg/mL) in all neuroprotection assays. The results (Table 2) show that *W. ovata* demonstrated notable protective effects, with EC_50_ values of 2.2 μg/mL for glutamate, 3.3 μg/mL for erastin, and 2.5 μg/mL for RSL3. Among the non-toxic species, *W. helenioides* demonstrated the strongest neuroprotective effects, with EC_50_ values of 5.7 μg/mL for glutamate, 9.6 μg/mL for erastin, and 9.7 μg/mL for RSL3. Similarly, *W. glabra*, *W. amplexicaulis* and *W. invenusta* exhibited strong protection across multiple oxytotic/ferroptotic stressors, with EC_50_ values ranging from 10.3 to 23.3 μg/mL. In contrast, *W. scabra, W. elata,* and *W. mollis* showed little or no protective effects, as their EC_50_ values exceeded 50 μg/mL for most stressors. While variability in neuroprotective efficacy among *Wyethia* species was observed, several species demonstrated strong protective effects, thereby supporting our working hypothesis.

### 3.4 Protection against energy loss in iodoacetic acid-induced neurotoxicity

Iodoacetic acid (IAA) induces cell death by disrupting glycolysis and causing oxidative stress, making it a useful model to study energy metabolism-related neurotoxicity. Protection against IAA toxicity was also evaluated in HT22 cells (Table 2). Among the tested species, *W. ovata* exhibited the strongest protection, with an EC_50_ value of 3.4 μg/mL. *W. glabra* (EC50 = 10.1 μg/mL), *W. helenioides* (EC_50_ = 11.1 μg/mL) and *W. amplexicaulis* (EC_50_ = 11.8 μg/mL) also showed strong protective effects. *W. invenusta* (EC_50_ = 15.6 μg/mL) and *W. mollis* (EC_50_ = 33.3 μg/mL) displayed moderate protection, while *W. scabra* and *W. elata* were not tested in this assay due to lack of protection against oxytosis/ferroptosis. Taken together, these results demonstrate that several *Wyethia* species show strong protection against energy metabolism-associated oxidative neurotoxicity, supporting the hypothesis that their traditional uses reflect genuine biochemical activities related to oxidative stress pathways.

### 3.5 Anti-inflammatory activity of *Wyethia* extracts in LPS-stimulated BV2 microglial cells

Neuroinflammation is a key contributor to neurodegenerative diseases, often mediated by activated microglia producing inflammatory molecules such as nitric oxide (NO). To assess the anti-inflammatory potential of *Wyethia* species, BV-2 microglial cells were stimulated with LPS (50 ng/mL) to induce nitric oxide (NO) production, a key marker of inflammation. The calculated EC_50_ values for each extract are summarized in Table 2. *W. scabra* and *W. elata* extracts were excluded from further anti-inflammatory evaluation because they did not demonstrate protective effects against oxytosis/ferroptosis in the HT22 cells.

**FIGURE 1:**
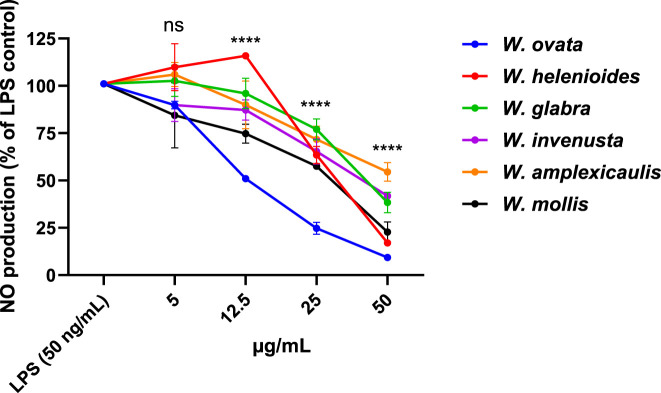
Inhibition of Lipopolysaccharide (LPS)-Induced Nitric Oxide Production by *Wyethia* species Extracts. Nitric oxide (NO) production in LPS-stimulated cells was normalized to cell density and expressed as a percentage relative to LPS control. Statistical analysis was performed using one-way ANOVA followed by post-hoc tests. Asterisks indicate significant differences between *Wyethia* extract-treated groups and the LPS control (50 ng/mL) (****p < 0.0001), while ‘ns’ denotes non-significant differences. Significance symbols at each concentration indicate that at least one extract showed a significant difference from the LPS control. Values represent the mean + SD, n = 3.

As shown in Figure 1, LPS treatment significantly increased NO production compared to the untreated control. Most of the tested *Wyethia* extracts exhibited a dose-dependent reduction in NO levels, with significant reduction observed at higher concentrations (p < 0.05 compared to LPS control). Among the tested species, *W. ovata, W. mollis* and *W. invenusta* exhibited the strongest NO-suppressing effects, particularly at 25 and 50 μg/mL.

Importantly, MTT analysis confirmed that the observed reduction in NO level was not due to cytotoxicity (data not shown), as none of the tested *Wyethia* extracts caused significant cell death at the tested concentrations. Notably, *W. ovata*, which was toxic to HT22 cells did not exhibit cytotoxic effects in BV-2 cells and demonstrated strong anti-inflammatory activity. These findings suggest that select *Wyethia* species possess promising anti-inflammatory properties by modulating NO production in activated microglia without compromising cell viability.

### 3.6 Intracellular glutathione (GSH) levels in HT22 cells treated with *Wyethia* extracts

**FIGURE 2:**
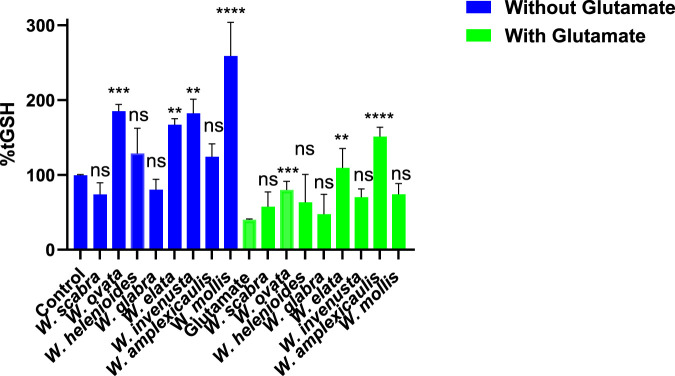
Effect of *Wyethia* species extracts (25 μg/mL, except *W. ovata* at 5 μg/mL) on intracellular glutathione (GSH) levels in HT22 cells in the presence and absence of glutamate (5 mM). Intracellular GSH levels were measured and expressed as percentage relative to the control. For the blue bars (without glutamate), each *Wyethia* extract’s GSH level was compared to the untreated control. For the green bars (with glutamate), GSH levels for each *Wyethia* extract were compared to glutamate-treated cells without extracts. Statistical significance is indicated as follows: ****p < 0.0001, ***p = 0.0005, **p < 0.01, and ns = not significant. Values represent mean ± SD, n = 3.

Glutathione (GSH) is a critical intracellular antioxidant that protects cells from oxidative damage and is a key factor in cellular defense mechanisms against oxytosis/ferroptosis, two forms of regulated cell death associated with neurodegeneration. Measuring GSH levels following treatment with *Wyethia* extracts provides insight into their potential to enhance antioxidant capacity and mitigate oxidative stress. As shown in Figure 2, treatment with *Wyethia* extracts alone resulted in variable effects on GSH levels. *W. elata*, *W. invenusta*, *W. mollis* and *W. ovata* significantly increased GSH levels compared to the control (p < 0.01 to p < 0.0001), with *W. mollis* showing the most pronounced increase (∼290% of control). In contrast, *W. scabra, W. helenioides* and *W. glabra* did not significantly alter GSH levels.

Glutamate treatment (5 mM) led to a substantial depletion of GSH (∼41% of control, p < 0.0001), confirming the expected reduction in GSH synthesis. Pre-treatment with *Wyethia* extracts provided varying degrees of protection. Notably, only *W. amplexicaulis* (p < 0.0001), *W. ovata* (p = 0.0005) and *W. elata* (p < 0.01) significantly maintained GSH levels in the presence of glutamate, while *W. scabra*, *W. glabra*, *W. invenusta* and *W. mollis* showed no significant effect compared to glutamate alone. These results suggest that *Wyethia* species, particularly *W. amplexicaulis* may enhance cellular antioxidant capacity by preserving intracellular GSH, which may contribute to their protective effects against oxidative stress.

### 3.7 Antioxidant activity measured by DPPH free radical scavenging assay

**FIGURE 3:**
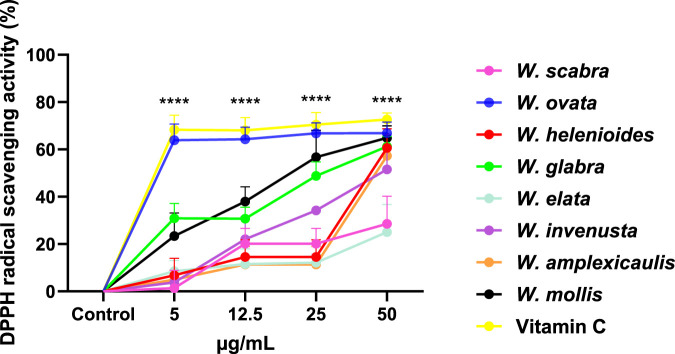
DPPH Radical Scavenging Activity of *Wyethia* Species Extracts at Different Concentrations. The antioxidant activity of *Wyethia* species extracts was assessed using the DPPH radical scavenging assay and expressed as a percentage of DPPH radicals scavenged relative to the control. Statistical analysis was performed using one-way ANOVA followed by post-hoc tests. Asterisks indicate concentrations at which at least one extract or the standard (Vitamin C) showed a significant difference compared to the control (****p < 0.0001). Values represent the mean + SD, n = 3.

The direct antioxidant potential of *Wyethia* species extracts was evaluated using the DPPH free radical scavenging assay, which measures the ability of compounds to neutralize free radicals and thus indicates their capacity to counteract general oxidative stress. Vitamin C was included as positive controls for comparison. As shown in Figure 3, the radical scavenging activity varied among the *Wyethia* species and within species increased in a concentration-dependent manner. All tested *Wyethia* species exhibited dose-dependent DPPH radical scavenging activity. Among the tested species, *W. ovata* and *W. mollis* exhibited the highest DPPH scavenging activity, approaching levels comparable to Vitamin C, whereas *W. scabra* and *W. elata* displayed the weakest activity across all concentrations. Other species, including *W. helenioides*, *W. invenusta*, *W. glabra*, and *W. amplexicaulis*, demonstrated moderate scavenging activity, with significant increases at higher concentrations (p < 0.0001). These findings suggest that certain *Wyethia* species possess strong antioxidant properties, likely due to their ability to neutralize free radicals.

### 3.8 Inhibition of lipid peroxidation by *Wyethia* extracts

**FIGURE 4:**
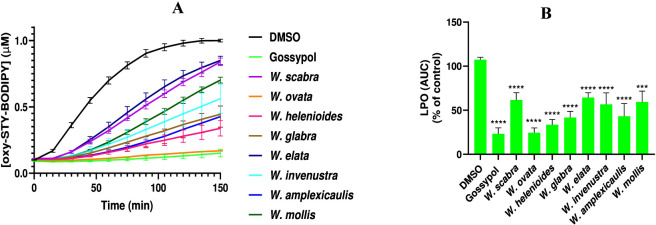
Inhibition of lipid peroxidation by *Wyethia* species extracts: time-dependent kinetics and area under the curve (AUC). **(A)** Time-dependent inhibition of lipid peroxidation ([oxy-STY-BODIPY], µM) by *Wyethia* species extracts in comparison to gossypol (positive control) and DMSO (negative control). Lipid peroxidation was monitored over 150 min. **(B)** Quantification of lipid peroxidation inhibition as the area under the curve (AUC), expressed as a percentage of the DMSO control. Statistical analysis was performed using one-way ANOVA followed by *post hoc* tests; significant differences between DMSO control and extract-treated groups are indicated (****p < 0.0001, ***p = 0.0005). Values represent the mean ± SD, n = 3.

Lipid peroxidation is a hallmark of oxytosis/ferroptosis, contributing to oxidative damage and cell death. The lipid peroxidation inhibitory activity of *Wyethia* species extracts was assessed using the oxidation of STY-BODIPY as a marker, with results expressed as the concentration of oxidized STY-BODIPY ([oxy-STY-BODIPY], µM) over time (Figure 4A) and as the area under the curve (AUC) relative to the control (DMSO) (Figure 4B). The kinetics of lipid peroxidation revealed distinct differences in the efficacy of *Wyethia* species extracts. The DMSO-treated control exhibited rapid and sustained lipid peroxidation, reaching the highest levels of [oxy-STY-BODIPY] (∼1.0 µM) over 150 min. In contrast, gossypol, a known lipid peroxidation inhibitor (Soriano-Castell et al., 2021a), maintained minimal oxidation levels throughout the assay, serving as a positive control.

Among the *Wyethia* species tested, *W. ovata*, *W. helenioides* and *W. amplexicaulis*, demonstrated the strongest inhibitory effects on lipid peroxidation, with a profile similar to gossypol. *W. invenusta*, and *W. glabra* also exhibited notable inhibition, although to a lesser extent. Conversely, *W. scabra*, *W. elata and W. mollis* showed weaker inhibition, with oxidation trends closer to the DMSO control.

The AUC analysis further confirmed these observations. Gossypol achieved near-complete inhibition of lipid peroxidation, with an AUC value close to zero. Among the *Wyethia* species, *W. ovata*, *W. helenioides* and *W. amplexicaulis* showed significant reductions in AUC compared to DMSO, indicating strong antioxidant activity (p < 0.001). In contrast, *W. scabra W. elata* and *W. mollis* had AUC values similar to DMSO, reflecting their weak inhibitory effects on lipid peroxidation. Moderate reductions in AUC were observed for *W. glabra* and *W. invenusta*, consistent with their intermediate activity. These results indicate that certain *Wyethia* species, particularly *W. helenioides*, possess strong lipid peroxidation inhibitory properties, which may contribute to their protective effects against oxytosis/ferroptosis. The variability in activity among species suggests differences in their phytochemical composition and antioxidant mechanisms.

### 3.9 Total phenolic content of *Wyethia* species

**FIGURE 5:**
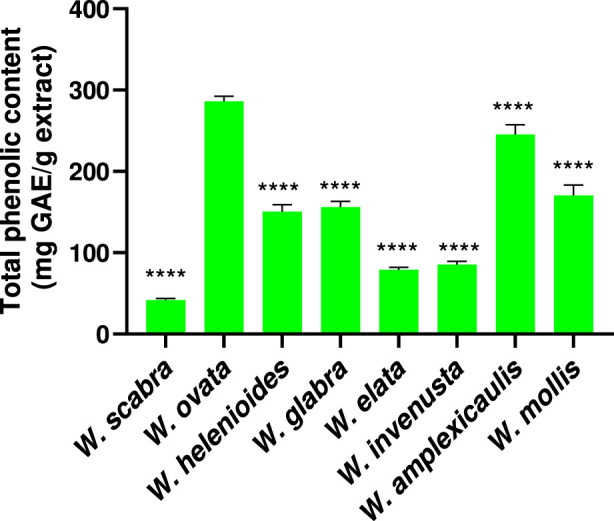
Total phenolic content was determined using the Folin–Ciocalteu assay and expressed as milligrams of gallic acid equivalents (mg GAE) per gram of extract. Values represent mean ± SD (n = 3). Statistical analysis was performed using one-way ANOVA followed by Dunnett’s multiple comparisons test, with *W. ovata* used as the reference group. Asterisks indicate significant differences compared to *W. ovata* (****p < 0.0001).

Phenolic compounds are important plant-derived antioxidants, which can contribute to neuroprotection by mitigating oxidative stress involved in oxytosis/ferroptosis. To quantify these compounds, the total phenolic content (TPC) of the *Wyethia* species was determined using the Folin-Ciocalteu assay with results expressed as milligrams of gallic acid equivalents (mg GAE) per gram of extract. The results revealed considerable variation in TPC among the different *Wyethia* species (Figure 5). *W. ovata* exhibited the highest TPC, measuring approximately 286 mg GAE/g extract, followed by *W. amplexicaulis* (≈245 mg GAE/g extract) and *W. mollis* (≈170 mg GAE/g extract). Moderate levels were observed in *W. glabra* and *W. helenioides* with values ranging between 120 and 160 mg GAE/g extract, which were higher than those of *W. elata* and *W. invenusta*. In contrast, *W. scabra* had the lowest phenolic content, with less than 50 mg GAE/g extract.

Taken together, the results of this study found that several extracts of *Wyethia* species exhibited robust antioxidant activity and significant neuroprotective and anti-inflammatory effects in the experimental models used. To provide a comprehensive comparison, Table 3 summarizes the DPPH radical scavenging activity (EC_50_), ability to increase GSH levels under glutamate-induced stress, lipid peroxidation inhibition, total phenolic content, neuroprotection across multiple pathways (including glutamate, erastin, RSL3 and IAA), and anti-inflammatory activity for each extract. The table highlights the distinct biochemical profiles and activities observed among the different species. This overview facilitates identification of species with the most promising bioactivity profiles.

**TABLE 3:** Summary of biochemical assay results for *Wyethia* species.

Plant name	DPPH EC_50_ (µg/mL)	GSH increase	LPO inhibition	Phenol content	Neuroprotection	Anti-inflammatory
*W. scabra*	>50	No	Low	Low	Low	Not tested
***W. ovata***	**7.3**	**Yes**	**High**	**High**	**High**	**High**
***W. helenioides***	**39.4**	**No**	**High**	**Moderate**	**Medium**	**Medium**
***W. glabra***	**27.3**	**No**	**Moderate**	**Moderate**	**Medium**	**Low**
*W. elata*	>50	Yes	Low	Low	Low	Not tested
*W. invenusta*	45.5	No	Moderate	Low	Low	Low
***W. amplexicaulis***	**44.3**	**Yes**	**High**	**High**	**Medium**	**Low**
*W. mollis*	21.3	No	Low	Moderate	Low	Medium

DPPH EC_50_ represents the concentration (µg/mL) required to reduce the DPPH, radical by 50%, where “>50 μg/mL” indicates the EC_50_ is greater than the highest concentration tested. GSH, Increase is denoted as “Yes” for a significant increase in glutathione (GSH) in HT22 cells treated with glutamate, and “No” for no significant increase. Lipid Peroxidation (LPO) Inhibition is classified as “High,” “Medium,” or “Low” based on the ability to inhibit lipid peroxidation in the BODIPY, assay. Phenol Content is categorized as “High,” “Medium,” or “Low” according to total phenolic content assay results. Neuroprotection is classified as “High,” “Medium,” or “Low” based on average EC_50_ values obtained from multiple neuroprotection assays including glutamate, erastin, RSL3 and IAA, toxicity, which evaluate diverse neuroprotective mechanisms including oxytosis/ferroptosis pathways. Anti-inflammatory Activity is categorized as “High,” “Medium,” or “Low” based on EC_50_ values derived from the anti-inflammatory assay. The term “Not tested” indicates that a specific assay was not performed for the particular species. Top-performing extracts are indicated in bold font for easy identification. Categories such as “High,” “Medium,” and “Low” represent relative activity levels based on experimental data.

**FIGURE 6:**
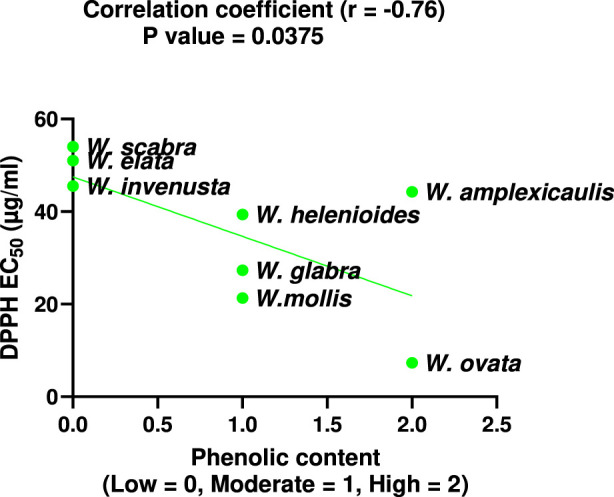
Correlation between total phenolic content and DPPH EC_50_ values for *Wyethia* species. Phenolic content was coded as Low = 0, Moderate = 1, High = 2. Lower DPPH EC_50_ values indicate higher antioxidant activity. A significant negative correlation was observed (Spearman r = −0.76, p < 0.05).

To further investigate the relationship between phenolic content and antioxidant capacity, a correlation analysis was performed. As shown in Figure 6, there is a strong, statistically significant negative correlation between total phenolic content and DPPH EC_50_ values across *Wyethia* species.

## 4 Discussion

This study demonstrated significant neuroprotective and anti-inflammatory effects of multiple *Wyethia* species extracts in *in vitro* models of oxytosis/ferroptosis, energy loss and inflammation. By utilizing herbarium specimens, we efficiently screened a diverse range of species without the need for fresh collections, leveraging the unique advantages of preserved botanical materials. Herbarium samples have recently gained recognition as valuable resources for drug discovery, enabling access to rare or geographically restricted taxa with well-documented provenance. For instance, Maher et al. (2020b) successfully identified neuroprotective and anti-inflammatory activities in herbarium specimens of the genus *Eriodictyon*. Our work builds on this approach by demonstrating the bioactivity of *Wyethia* species, supporting their further investigation as candidates for therapeutic development in neurodegenerative and inflammatory diseases. Herbarium specimens offer valuable botanical material for screening, with compound classes like flavonoids and phenolic acids generally stable over long periods. While some volatile or labile compounds may degrade, no significant phytochemical degradation was observed in herbarium specimens studied previously in our lab (Maher et al., 2020b). These findings support the reliability of herbarium samples for bioactivity assessment.

Selected *Wyethia* species, including *W. ovata, W. glabra*, *W. helenioides*, *W. invenusta*, and *W. amplexicaulis* demonstrated significant protection of HT22 cells from glutamate, erastin and RSL3 induced toxicity, which are models of oxytosis/ferroptosis. In addition, these species also provided significant protection against IAA-induced toxicity, which represents a distinct mechanism of cell death. The observed neuroprotection aligns with findings in previous studies on plant-derived polyphenols, which have been shown to mitigate oxidative stress by counteracting glutathione depletion and maintaining mitochondrial function (Fischer et al., 2019; Cui et al., 2020; Taïlé et al., 2020). The ability of these *Wyethia* species to protect across multiple oxidative stressors is particularly compelling, as evidenced by their low EC_50_ values (2.2–23.3 μg/mL). This suggests that their protective effects may not be confined to a single mechanism but could reflect a broader capacity to stabilize redox homeostasis and preserve cellular energy production.

Interestingly, *W. ovata* exhibited a dose-dependent dual effect, providing neuroprotection at low concentrations, while showing cytotoxicity at higher doses in HT22 cells. This biphasic response may be explained by hormesis, a phenomenon where low doses of bioactive compounds induce adaptive protective responses, whereas higher doses cause toxicity. Sesquiterpene lactones, known constituents of *Wyethia* species, have been reported to exhibit such concentration-dependent dual activities (da Silva et al., 2021) This observation is critical in interpreting the neuroprotective data and suggests a narrow therapeutic window for certain extracts, which warrants further mechanistic investigation.

The anti-inflammatory activity of *Wyethia* extracts further supports their therapeutic potential, as several species effectively inhibited nitric oxide (NO) production in LPS-stimulated BV-2 microglial cells. In particular, *W. ovata*, *W. mollis*, and *W. amplexicaulis* exhibited strong, dose-dependent inhibition of NO production without inducing cytotoxicity. These results are consistent with previous studies demonstrating that plant-derived compounds can modulate inflammation by targeting key signaling pathways such as NF-κB and MAPK, which regulate the expression of pro-inflammatory mediators (Saleh et al., 2021; Kang et al., 2023; Nakadate et al., 2025). Given the role of chronic inflammation in neurodegeneration, these dual neuroprotective and anti-inflammatory effects underscore the therapeutic promise of *Wyethia* species.

Assessment of intracellular glutathione (GSH) levels revealed partial restoration with *W. amplexicaulis*, *W. ovata*, and *W. elata*. Preservation of GSH is significant, given that its depletion is a hallmark of oxytosis/ferroptosis and its restoration is a key neuroprotective strategy (Maher et al., 2020b). Interestingly, despite elevating GSH, *W. elata* did not protect cells from glutamate, erastin, or RSL3 toxicity, suggesting that GSH restoration alone may be insufficient for full neuroprotection. This discrepancy likely reflects differences in phytochemical profiles and indicates that additional mechanisms contribute to effective protection.

The DPPH radical scavenging results demonstrate strong antioxidant activity for *W. ovata* and *W. mollis.* This likely reflects their high phenolic content, known to donate hydrogen atoms to stabilize free radicals. Most other *Wyethia* species exhibited moderate scavenging activity in a dose-dependent manner, consistent with the typical behavior of plant-derived antioxidants in DPPH assays (Sagbo et al., 2017; Gulcin and Alwasel, 2023). Notably, *W. scabra* and *W. elata*, which displayed weak DPPH activity also showed little to no neuroprotective effects in the neuroprotection assays. This correlation suggests that direct radical scavenging may contribute significantly to the neuroprotective potential of *Wyethia* species. The lack of both antioxidant and neuroprotective activity in *W. scabra* and *W. elata* highlights the importance of free radical neutralization as a key mechanism in mitigating oxidative stress-related toxicity.

The lipid peroxidation data provide compelling evidence that specific *Wyethia* species also possess a strong ability to inhibit membrane lipid oxidation. Notably, *W. ovata*, *W. helenioides*, *W. amplexicaulis*, and *W. invenusta* showed strong suppression of STY-BODIPY oxidation, with profiles resembling the known lipid peroxidation inhibitor gossypol. These findings suggest that these species contain bioactive compounds capable of directly scavenging lipid radicals or disrupting chain reactions involved in oxidative membrane damage. Thus, the lipid peroxidation inhibitory capacity of these extracts likely plays a central role in their neuroprotective effects observed against glutamate, erastin and RSL3-induced cell death. The variation in activity among species ranging from strong inhibition with *W. ovata* to weak or negligible effects with *W. scabra* and *W. elata*, highlights interspecies differences in anti-lipid peroxidation properties, likely driven by distinct phytochemical profiles.

The differences observed between the DPPH radical scavenging and lipid peroxidation inhibition assays likely reflect the distinct antioxidants each assay detects. DPPH mainly measures the ability to neutralize hydrophilic radicals, while lipid peroxidation assays assess the ability to neutralize lipophilic radicals. This likely explains why some extracts act strongly in one assay but weakly in the other as the effects will depend on the distinct array of compounds present in each extract. These differences further support the use of multiple assays for a full antioxidant profile. The observed variation in TPC across *Wyethia* species highlights differential polyphenol accumulation, with *W. ovata* and *W. amplexicaulis* exhibiting the highest TPC. While *W. ovata* displayed strong antioxidant activity in both DPPH and lipid peroxidation assays, *W. amplexicaulis* showed strong lipid peroxidation inhibition but moderate DPPH radical scavenging activity. Several other species, including *W. helenioides*, *W. glabra*, and *W. mollis*, demonstrated moderate TPC and varying levels of antioxidant capacity. These results support the multifunctional antioxidant role of phenolics described in the literature, as only *W. ovata* exhibited both high total phenolic content and potent antioxidant activity across all assays. This indicates that, in *W. ovata*, polyphenols likely serve as effective radical scavengers and inhibitors of lipid peroxidation, as previously reported (Aryal et al., 2019; Osman et al., 2020; Andrés et al., 2024). However, not all of the *Wyethia* species showed significant neuroprotection in the oxytosis/ferroptosis models indicating that polyphenol content alone is not a reliable predictor of neuroprotective activity. Notably, *W. invenusta,* despite an intermediate TPC, demonstrated significant neuroprotection, suggesting non-phenolic compounds (terpenoids, alkaloids) may drive its effects. For instance, oleanolic acid, a terpenoid found in some plants, correlates with antioxidant activity in other species, while sulfur-containing compounds could modulate oxytosis/ferroptosis by acting as precursors for glutathione synthesis or by directly inhibiting lipid peroxidation, thereby protecting cells from death (Osman et al., 2020; Ortiz-Mendoza et al., 2023). Indeed, sesquiterpene lactones are common in many Asteraceae plants and are characterized by reactive α-methylene-γ-lactone groups that interact with cellular proteins and modulate pathways relevant to antioxidants, NF-κB inhibitory, and cytotoxic activities (Matos et al., 2021). Although their presence in *Wyethia* species has not been extensively characterized, they could contribute to the observed bioactivity and toxicity patterns. These results suggest that, beyond polyphenols, other compound classes present in *Wyethia* extracts could play a significant role in the observed neuroprotective and antioxidant activities. Conversely, *W. scabra* and *W. elata* showed low TPC and limited neuroprotective activity. In *W. elata*, enhancement of GSH alone was insufficient to block oxytosis/ferroptosis consistent with its very weak inhibition of lipid peroxidation and lack of DPPH radical scavenging activity. Therefore, effective protection against oxytosis/ferroptosis may require the combined action of multiple pathways. These findings underscore TPC as an important, but not exclusive, determinant of the bioactivity of *Wyethi*a.

Overall, the multifaceted protection observed likely arises from a complex interplay of antioxidant activity, glutathione preservation, and anti-inflammatory effects across species, highlighting their diverse chemical profiles. This diversity, coupled with the emerging relevance of oxytosis/ferroptosis and inflammation in neurodegeneration (Soriano-Castell et al., 2021b; Currais et al., 2025), positions *Wyethia* species as promising neuroprotective agents.

Given the complex nature of neurodegenerative diseases involving oxidative stress, oxytosis/ferroptosis, and inflammation, whole *Wyethia* extracts may provide advantages over isolated compounds for therapeutic use. The observed bioactivities suggest a plausible mechanism of action centered on multifaceted oxidative stress modulation through direct radical scavenging, inhibition of lipid peroxidation cascades and preservation of glutathione homeostasis. The diverse bioactive compounds in the extracts could work synergistically to enhance neuroprotective effects by targeting multiple convergent pathways. Minor constituents may further improve efficacy and bioavailability. Therefore, using whole extracts leverages natural phytochemical interactions, potentially offering more effective and safer strategies to combat neurodegeneration. Importantly, the methodological approach applied here, which prioritizes plants traditionally used by native peoples, is significantly facilitated by the utilization of herbarium samples. Herbarium collections provide invaluable, well-documented plant material that greatly enhances the feasibility and depth of ethnopharmacological studies, reinforcing the bridge between traditional knowledge and modern therapeutic discovery.

## 5 Conclusion

This study highlights the neuroprotective potential of selected *Wyethia* species mediated through multiple mechanisms including antioxidant activity, lipid peroxidation inhibition, glutathione preservation, and anti-inflammatory effects. However, notable interspecies variability highlights the importance of comprehensive phytochemical profiling to identify the most promising candidates. The interspecies differences likely reflect unique phytochemical profiles and mechanisms of action. Importantly, this study underscores the value of an ethnopharmacological approach that integrates the use of medicinal plants historically employed by native peoples with modern extraction from well-documented herbarium specimens. This combination facilitates rigorous investigation of bioactive extracts, as exemplified by the positive anti-oxytotic/ferroptotic and anti-inflammatory effects observed here. This methodological framework, leveraging both traditional knowledge and herbarium resources, advances the identification and validation of promising neuroprotective botanical candidates, supporting future drug discovery efforts against neurodegenerative diseases. Future work will focus on bioassay-guided fractionation and LC-MS profiling to identify active compounds in the most effective extracts which was beyond the scope of this initial bioactivity-driven screening study. Future *in vivo* validation is also planned in order to establish therapeutic potential.

## Data Availability

The original contributions presented in the study are included in the article/supplementary material, further inquiries can be directed to the corresponding author.
